# Regulation of phenylacetic acid degradation genes of *Burkholderia cenocepacia *K56-2

**DOI:** 10.1186/1471-2180-9-222

**Published:** 2009-10-18

**Authors:** Jason NR Hamlin, Ruhi AM Bloodworth, Silvia T Cardona

**Affiliations:** 1 Department of Microbiology, University of Manitoba, Winnipeg, Manitoba, Canada; 2 Current address : Department of Neurology and Neurosurgery, Montreal Neurological Institute, McGill University, Montreal, Quebec, Canada

## Abstract

**Background:**

Metabolically versatile soil bacteria *Burkholderia cepacia *complex (Bcc) have emerged as opportunistic pathogens, especially of cystic fibrosis (CF). Previously, we initiated the characterization of the phenylacetic acid (PA) degradation pathway in *B. cenocepacia*, a member of the Bcc, and demonstrated the necessity of a functional PA catabolic pathway for full virulence in *Caenorhabditis elegans*. In this study, we aimed to characterize regulatory elements and nutritional requirements that control the PA catabolic genes in *B. cenocepacia *K56-2.

**Results:**

Translational fusions of the PA degradation gene promoters with *eGFP *were constructed and introduced in *B. cenocepacia *K56-2. eGFP expression was observed when the reporter strains were grown in minimal media containing glycerol and PA or other compounds expected to proceed through the PA pathway, and in synthetic CF medium (SCFM). Addition of succinate or glucose to the PA containing medium repressed eGFP expression. To show that *BCAL0210*, a putative TetR-type regulator gene encodes a regulator for the PA genes in *B. cenocepacia*, we developed a *BCAL0210 *insertional mutant reporter strain. Results show that these strains exhibit fluorescence regardless of the presence of PA in the culture.

**Conclusion:**

The PA catabolic genes of *B. cenocepacia *K56-2 are induced by PA and other related compounds, are negatively regulated by PaaR (named herein), a TetR-type regulator, and are subjected to catabolic repression by glucose and succinate. As the PA catabolic pathway of *B. cenocepacia *appears to be induced during growth in synthetic cystic fibrosis medium (SCFM), further research is necessary to determine the relevance of this pathway in CF-like conditions and in other host-pathogen interactions.

## Background

The *Burkholderia cepacia *complex (Bcc) is a group of Gram negative bacteria that comprises at least fifteen taxonomically related species [1,2]. Bcc strains occupy multiple niches from soil to humans as they have emerged as opportunistic pathogens in patients with cystic fibrosis (CF), chronic granulomatous disease, and other medical conditions associated with a compromised immune system [1,3]. *Burkholderia *species have evolved large genomes that allow them to deal with a variety of nutrient sources, predation and competition. The three chromosomes of *B. cenocepacia*, one of the most common species found in CF patients [4], encode a broad array of catabolic functions. Yet, the contribution of these metabolic capacities to colonization and survival in the host has not been established.

The phenylacetic acid (PA) catabolic pathway is the central route where catabolism of many aromatic compounds converge and are directed to the Krebs cycle [5]. It comprises of four steps, namely the PA-CoA ligation-activation performed by PaaK [6], the hydroxylation step for which the PaaABCDE enzymatic complex is responsible [7], the enoyl-CoA isomerization/hydration, ring opening performed by PaaG and PaaZ, [8], and the β-oxidation step carried out by PaaF and PaaH, [8].

Previously, we initiated the functional characterization of the PA catabolic pathway of *B. cenocepacia *K56-2 [9] and demonstrated that interruption of putative PA-CoA ring hydroxylation activity, but not the lower steps of PA degradation, resulted in an attenuated pathogenic phenotype in the *Caenorhabditis elegans *model of infection. Here, we report that the PA catabolic genes of *B. cenocepacia *K56-2 are induced by PA, are negatively regulated by PaaR, a TetR-type regulator and are subjected to catabolic repression by glucose and succinate.

## Results

### Translational reporter plasmids containing PA catabolic gene promoters are responsive to PA and related compounds

**Figure 1:**
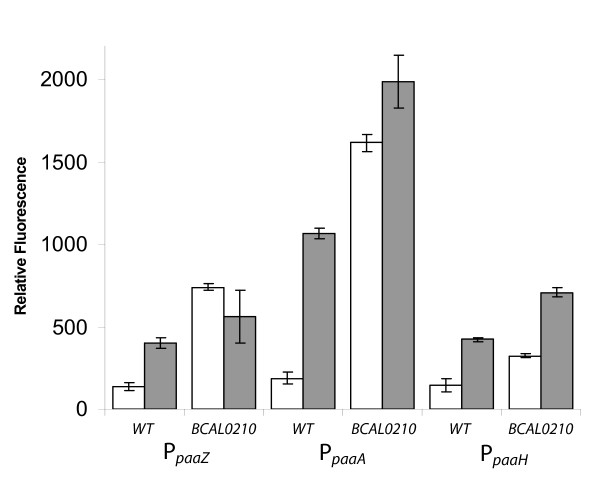
**Phenylacetic Acid Responsive PA reporters**. *B. cenocepacia *K56-2 (WT) or JNRH1 (BCAL0210) containing *eGFP *translational reporters P_*paaZ*_, P_*paaA *_and P_*paaH *_were grown for 18 hours in M9 minimal media supplemented with glycerol (white bars) or PA and glycerol (grey bars). Relative fluorescence was determined as described in methods. Data represent the mean from three independent experiments, with error bars signifying standard deviations.

The PA degradation genes are arranged in three separate clusters in *B. cenocepacia*, namely *paaABCDE, paaFZJGIJK1 *and *paaHK2*, where the *paaF *gene is divergently orientated from the *paaZJGIJK1 *cluster [9]. To evaluate whether the upstream regions of the PA catabolic genes contained PA-inducible promoters, translational *eGFP *reporter plasmids containing DNA fragments upstream of the first gene of each of the three PA clusters: *paaA*, *paaZ *and *paaH *were constructed and introduced into *B. cenocepacia *K56-2. Previous results showed that eGFP is expressed and remains stable in *B. cenocepacia *[10]. Cells containing reporter plasmids with the *paaA*, *paaH*, and *paaZ *promoters (P_*paaA*_, P_*paaH*_, and P_*paaZ *_respectively) fused to the *eGFP *gene, exhibited increased fluorescence when grown in minimal media containing glycerol with PA in comparison with those grown in minimal media containing glycerol without PA (Figure 1). eGPF expression from P_*paaA *_was 5.7 fold higher when grown with PA compared to glycerol, while the ones from P_*paaH *_and P_*paaZ *_were each 2.9 fold higher.

**Figure 2:**
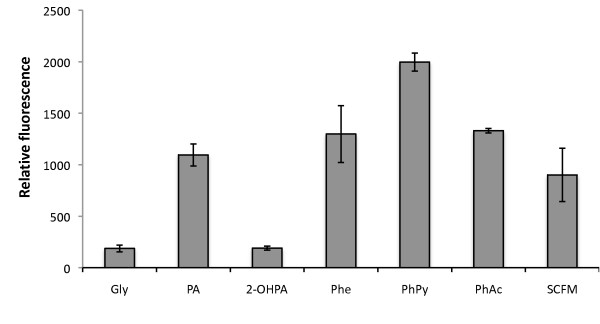
**Activity of P*paaA *as a result of growth in M9 minimal media with different carbon sources**. *B. cenocepacia *K56-2 (WT) containing *eGFP *translational reporters P_*paaA *_were grown for 18 hours in synthetic cystic fibrosis medium (SCFM) or M9 minimal media supplemented with various carbon sources. Gly, glycerol; PA, phenylacetic acid; 2-OHPA, 2-hydroxy-phenylacetic acid; Phe, L- phenylalanine; PhPy, phenylpyruvate; PhAc, phenylacetamide. Relative fluorescence was determined as described in methods. Data represent the mean from three independent experiments, with error bars signifying standard deviations.

According to the KEGG database [11-13] we expected phenylalanine, phenylacetamide and phenylethylamine to be degraded through the PA catabolic pathway in *B. cenocepacia *AU1054. To determine if these aromatic carbon sources induce the PA degradation pathway in *B. cenocepacia *K56-2, cells containing the P_*paaA *_reporter were grown in media containing these carbon sources. eGFP expression similar to the one shown with PA was observed with phenylalanine, phenylpyruvate or phenylacetamide (Figure 2). On the contrary, 2-hydroxy-phenylacetic acid did not induce eGFP expression, in accordance with this compound not being a true intermediate of the pathway [6].

In addition, we sought to determine whether the PA genes were activated in response to Synthetic Cystic Fibrosis Medium (SCFM), a chemically defined medium formulated according to the contents of CF sputum [14]. Our results show that P_*paaA *_reporter activity increases approximately 5-fold when cells are grown in SCFM (Figure 2).

### The PA catabolic genes are subject to catabolic repression by TCA intermediates and sugars

**Figure 3:**
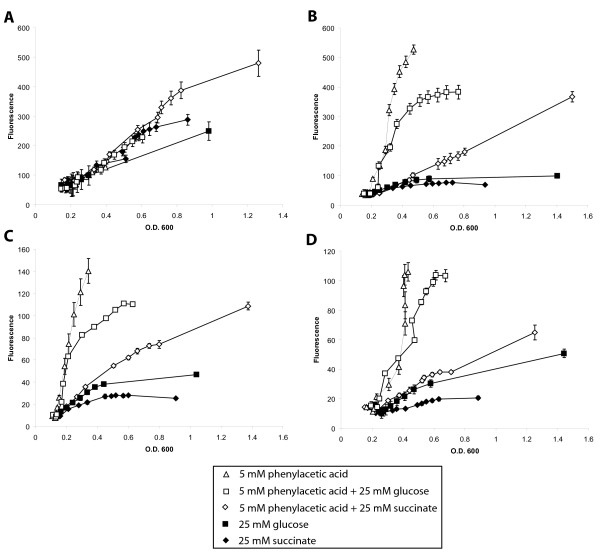
**Phenylacetic acid genes are subject to Carbon Catabolite Repression**. *B. cenocepacia *K56-2 containing *eGFP *translational fusions with the *dhfr *promoter (A), P_*paaA *_(B), P_*paaZ *_(C), and P_*paaH *_(D) were grown for 13 hours in M9 minimal media supplemented with the indicated carbon sources. Error bars represent the standard deviation of three independent cultures.

Aromatic compound degradation is subject to catabolic repression in the presence of more readily usable carbon sources in other bacteria [15-17]. Therefore, the possible catabolic repression exerted by succinate and glucose was investigated. Strains containing the reporters P_*paaA*_, P_*paaZ *_and P_*paaH *_or the plasmid pJH1 were grown in minimal medium containing PA with or without the additional carbon source and analyzed at one-hour intervals (Figure 3). *B. cenocepacia *K56-2 harbouring pJH1 was used as a control as the *dhfr *promoter is constitutive in *Burkholderia *species [10,18]. Figure 3A shows that fluorescence increased linearly with optical density in the media types tested, indicating the rate of eGFP expression does not change during growth with each of the conditions in *B. cenocepacia*. Initially, the levels of eGFP expression were not affected with the different carbon sources, although at optical densities above 0.6, fluorescence varied slightly depending on the different carbon sources used. Catabolic repression by glucose on the PA-inducible eGFP expression was observed in cells harbouring P_*paaA*_, at approximately an O.D_600 _of 0.3 where a shift in the slope towards steady levels of fluorescence, suggesting lack of *de novo *eGFP synthesis, was observed (Figure 3B). The same effect was observed with reporters P_*paaZ *_and P_*paaH *_(Figure 3C and 3D respectively). This is contrasted with cells grown in succinate, which exhibited strong silencing of eGFP expression at all cell densities (Figure 3B-D). We concluded that glucose and succinate exert catabolic repression of the PA degradation pathway.

### Insertional mutagenesis of *BCAL0210 *results in increased expression of PA-inducible genes

**Figure 4:**
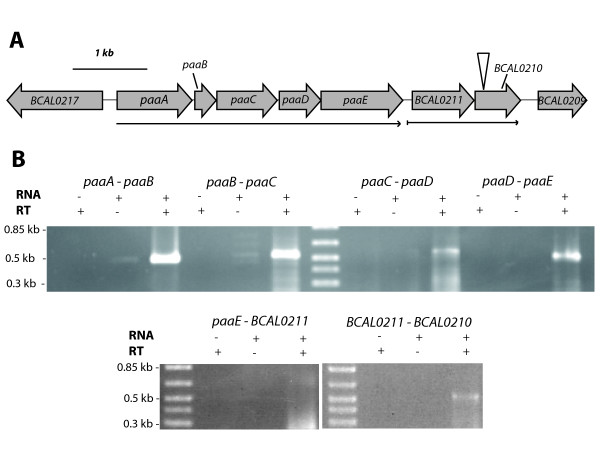
**Genetic and transcriptional organization of the *paaABCDE *and *BCAL0211-BCAL0210 *gene clusters**. A) Fragment of chromosome 1 of *B. cenocepacia *J2315 containing the *paaABCDE *and *BCAL0211-0210 *gene clusters. The vertical arrow indicates the location of the inserted pJH9. Horizontal arrows represent transcriptional units (see B). B) RT-PCR analysis of the intergenic regions of the *paaABCDE *and *BCAL0211-0210 *gene clusters. 500 bp RT-PCR amplified DNA bands correspond to intergenic regions.

Located 128 bp downstream of the *paaABCDE *gene cluster and oriented in the same direction are genes *BCAL0211 *and *BCAL0210 *(Figure 4A). *BCAL0211 *is predicted to encode a 273 amino acid protein containing a conserved domain of unknown function (DUF1835 superfamily) while BCAL0210 was annotated as a TetR family regulatory protein. Results of our BLAST search indicated the N-terminal region of BCAL0210 protein shows 60% similarity to AcrR (Expect value = 5e-7), which is a TetR-like regulator of a multi-drug efflux pump of *E. coli *[19-21]. Given that a regulator protein homologous to PaaX, the GntR-type transcriptional regulator of PA degradation in *E. coli *[22] is not encoded in *B. cenocepacia *J2315 genome, we hypothesized that the *BCAL0210 *gene encoded the regulator of PA catabolism in *B. cenocepacia*. The effect of the loss of *BCAL0210 *function on the regulation on the PA genes was determined by insertional mutagenesis of the *BCAL0210 *gene to create the strain JNRH1. Reporter plasmids containing the P_*paaA*_, P_*paaH *_and P_*paaZ *_promoters were then conjugated into strain JNRH1 and eGFP expression was assessed in the presence and the absence of PA. Results show that these strains exhibit increased fluorescence regardless of the presence of PA in the culture (Figure 1). This PA independent activity suggests that *BCAL0210 *encodes for a negative regulator, whose regulatory ability is abolished in the JNRH1 mutant. Interestingly, eGFP expression driven by the P_*paaA *_and P_*paaH *_promoters in JNRH1 was higher in the presence of PA than in reporter strains grown with glycerol only (Figure 1) suggesting a BCAL0210 independent induction of gene expression in the presence of PA.

In order to determine if *paaABCDE *and *BCAL0211*-*BCAL0210 *were part of the same transcriptional unit, a transcriptional analysis was performed. Total RNA was isolated from *B. cenocepacia *cells grown with LB containing 1 mM PA and subjected to RT-PCR using specific primers. Results show that the *paaA, paaB, paaC, paaD *and *paaE *genes are contained on a single transcript and are thus co-regulated at the transcriptional level (Figure 4B). Primers were unable to generate an amplicon between *paaE *and *BCAL0211 *although an amplicon was generated between *BCAL0211 *and *BCAL0210*, indicating that they are located on the same transcript. Taken together these results demonstrate that *paaABCDE *and *BCAL0211-BCAL0210 *are two separate transcriptional units.

### A conserved Inverted Repeat is necessary for negative control of P_*paaA*_

**Figure 5:**
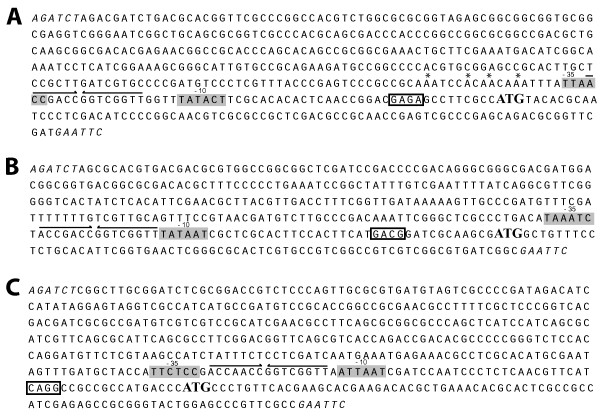
**Conserved inverted repeat detected in the *paaA*, *paaZ *and *paaH *promoters**. DNA Sequences of P_*paaA*_, (A), P_*paaH*_, (B), and P_*paaZ*_, (C), cloned in pJH2. Predicted start codon is highlighted in bold. Putative ribosome binding site is boxed; predicted -10 and -35 regions are highlighted in grey. The detected conserved inverted repeats are underlined with arrows. A putative UP element is indicated with asterisks.

Examination of upstream DNA sequences of the PA gene clusters identified near perfect 15 bp inverted repeat (IR) sequences located between the putative -10 and -35 core promoter signals (Figure 5) that resembled operator sites of a TetR regulatory protein [21]. In order to validate the IR sequences found in PA gene promoters as the operator sites of BCAL0210, translational fusion plasmids containing mutations in the *paaA *IR were created. We hypothesized that the sequence is a motif recognized by a TetR-like transcriptional regulator due to it being a dual overlapping inverted repeat, similar to the QacR operator [21].

Using plasmid mutagenesis with primers containing mismatched mutations on the 5' ends (Additional file 1) that annealed to plasmid pJH7 containing the P_*paaA *_reporter we generated plasmids pJH10, pJH11 and pJH12 (Table 1). The plasmid pJH10 contains 14 mismatch mutations replacing nearly the entire IR within the *paaA *promoter. Plasmids pJH11 and pJH12 contain the mutations in the upstream or downstream half of the IR respectively. These plasmids were then transferred to *B. cenocepacia *K56-2 by triparental mating. Reporter strains were grown in minimal media supplemented with glycerol or PA. Cells harbouring plasmids pJH10, PJH11 and pJH12 exhibited higher levels of relative fluorescence in comparison with K56-2/pJH7 when grown with glycerol, demonstrating that the sequence is indeed required for negative control of *paaA *promoter activity (Table 2).

**Table 1:** Bacterial Strains and Plasmids

Strain or plasmid	Features	Reference or source
*B. cenocepacia *strains		
		
K56-2 (LMG18863)	ET12 clone related to J2315, CF clinical isolate	[43]
JNRH1	K56-2*BCAL0210*::pJH9, Tp^r^	This study
*E. coli *strains		
DH5α	F^-^, ϕ 80 *lac*ZΔM15 *endA1 recA1 hsdR*17(r_K_^-^m_K_^+^)*supE*44 *thi-*1 Δ*gyrA*96 (*ΔlacZYA*-*arg*F)U169 *relA*1	Invitrogen
SY327	*araD *Δ(*lac pro*) *argE *(Am) *recA*56 Rif^r ^*nalA *λ *pir*	[40]
		
Plasmids		
		
pGPΩTp	*ori *_r6K_, ΩTp^r ^*mob+*	[27]
pRK2013	*ori*_colE1_, RK2 derivative, Km^r ^*mob*^+ ^*tra*^+^	[42]
pJH9	pGPΩTp, internal fragment from *BCAL0210*	This study
pJH1	pap20, *eGFP*	[9]
pJH2	pJH1, *eGFP *replaced promoter with multiple cloning site	This study
pJH5	pJH2, *BCAL0211*promoter region (P_*BCAL0211*_)	This study
pJH6	pJH2, *paaZ *promoter region (P_*paaZ*_)	This study
pJH7	pJH2, *paaA *promoter region (P_*paaA*_)	This study
pJH8	pJH2 *paaH *promoter region (P_*paaH*_)	This study
pJH10	pJH7, ACCGACCGGTCGGT → TAGATGTATCTCAG	This study
pJH11	pJH7, ACCGACCGGTCGGT → TAGATGTGGTCGGT	This study
pJH12	pJH7, ACCGACCGGTCGGT → ACCGACCATCTCAG	This study

Cm, chloramphenicol; Km, kanamycin; Tp, trimethoprim. Arrows represent changes introduced to indicated sequences.

Because PaaX is involved in the regulation of upstream pathways of PA catabolism in other microorganisms through binding a conserved PaaX box [23,24] we searched for the consensus IR sequence in the genome of *B. cenocepacia*. A position weight matrix (PWM) [25] of the conserved IR present in the promoter regions of the *paaA, paaH *and *paaZ *plus the divergent promoter of *paaF *and *BCAL0211 *was constructed (Additional file 2) and used to search the entire genome sequence of *B. cenocepacia *J2315. The coordinate positions of sequences detected up to a cut off score of 17.0 are listed (Additional file 3). The top scores for the search were the ones for the *paaZ, paaF*, *paaA *and *paaH *inverted repeats while BCAL0211 IR scored lower at 12.0. Other sequences with scores that ranked from 18.41 to 17.37 did not locate in putative promoters or between -10 and -35 regions, likely representing false positives. We concluded that the 15 bp IR sequences are specific to the PA catabolic gene clusters.

## Discussion

In contrast to what has been observed in *E. coli *and *Pseudomonas putida *[5], the PA genes of *B. cenocepacia *K56-2 are organized into three gene clusters. We hypothesize that this arrangement may allow regulation of gene expression at different levels. The observation that eGFP expression driven by P_*paaA *_is roughly 3-fold stronger than either the P_*paaH *_or P_*paaZ *_promoters (Figure 1) is suggestive of a higher requirement for the product of the PaaABCDE enzymatic complex than the other intermediates. This could be simply due to the optimal kinetic coupling between the different steps or that the product of the ring hydroxylation complex is used in a second pathway with a yet unknown biological function. The presence of a poly(A) tract upstream of the *paaA *-35 element (Figure 5A) that resembles an UP element [26] may likely account for the increased activity.

Our results also show that *BCAL0210 *is necessary for repression of PA dependent activity of the *paaA, paaH *and *paaZ *gene promoters (Figure 1). Therefore, *BCAL0210 *(PaaR) encoding for a TetR-type transcriptional regulator is involved in negative regulation of the PA catabolic genes. Since a conserved inverted repeat DNA sequence is necessary for PA negative control of *paaA *gene expression (Table 2), we hypothesize that BCAL0210 binds the IRs located in the core promoter of the *paaA*, *paaZ *and *paaH *genes to negatively regulate transcription of the PA catabolic genes. It should be noted however, that the insertional mutagenesis system used to produce JNRH1 introduces polar mutations [27]. Although the possibility of polar effects on genes downstream BCAL0210 cannot be ruled out, the downstream gene *BCAL0209*, encoding a putative GNAT family acetyl transferase located several hundred base pairs downstream of *BCAL0211 *makes the possibility of polar effects unlikely. On the other hand, *BCAL0211 *and *BCAL0210 *are located on the same transcript (Figure 4) and thus are co-regulated at the transcriptional level. TetR-type proteins are known to regulate their own transcription by self-repression [28]. Currently it is unknown if the conserved IR located in the DNA leader sequence of the *BCAL0211 *gene may be involved in regulation of this gene cluster. Whether *BCAL0211*, which encodes for a protein of unknown function (DUF1835) is involved in some fashion in the regulation of the PA genes remains to be determined.

**Table 2:** Activity of P*paaA *and IR mutated derivatives as a result of growth in M9 minimal media containing glycerol or PA.

Strain/plasmid	Mean fluorescence/O.D.600 ± SD with indicated carbon sources	
	
	Gly	PA
K56-2/pJH7	187 ± 33	1096 ± 107
K56-2/pJH10	1579 ± 10	1062 ± 15
K56-2/pJH11	1345 ± 111	1026 ± 52
K56-2/pJH12	2159 ± 111	1503 ± 60

*B. cenocepacia *K56-2 containing *eGFP *translational reporters P_*paaA *_were grown for 18 hours in M9 minimal media supplemented with glycerol or PA. Relative fluorescence was determined as described in methods. Data represent the mean from three independent experiments, ± one standard deviation.

Catabolic repression of aromatic compound degradation by TCA intermediates and glucose has been described in the β-proteobacterium *Acidovorax sp*. [29], and *P. putida *[15] respectively. In accordance with these data we found that the PA catabolic pathway of *B. cenocepacia *K56-2 is subject to catabolic repression by glucose and succinate (Figure 3). Interestingly, P_*paaA *_is induced after 18 h of growth in SCFM probably as a result of the presence of phenylalanine (Figure 2). This observation is consistent with the recently reported *B. cenocepacia *global gene expression response to SCFM, which shows the induction of the PA catabolic pathway [30]. Whether this finding is relevant for pathogenesis of Bcc in the CF lung environment remains an unexplored point of interest.

## Conclusion

We show that the PA gene promoters are responsive to PA, SCFM, and other compounds expected to proceed via the PA pathway. We also show the PA gene promoters are negatively regulated by PaaR, a TetR-type regulator, and are subjected to catabolic repression by succinate and glucose.

## Methods

### Bacterial strains, nematode strains and growth conditions

Bacterial strains and plasmids are listed in Table 1. *B. cenocepacia *K56-2 was grown at 37°C in Luria Bertani (LB) or M9 minimal medium with 5 mM PA or 25 mM of the indicated carbon sources, supplemented as required, with 100 μg/ml trimethoprim (Tp), 50 μg/ml gentamicin (Gm) and 200 μg/ml chloramphenicol (Cm). *E. coli *was grown at 37°C in LB medium supplemented with 50 μg/ml Tp, 40 μg/ml kanamycin (Km) or 20 μg/ml Cm.

### Reporter activity assays

96-well microplates containing 150 μl of M9 minimal media supplemented with indicated carbon source(s) were inoculated with 2 μl from an overnight culture grown in LB, washed with PBS and adjusted to an O.D. 600 of 2.0 with M9 minimal salts. 96-well microplates were incubated at 37°C with shaking at 200 rpm. eGFP protein has excitation/emission wavelengths of 488/509 [31]. Relative fluorescence, defined as the ratio between arbitrary fluorescence and optical density at 600 nm (O.D.600) was measured with a Biotek Synergy 2 plate reader, using excitation 485/20 and emission 528/20 filter sets. O.D. 600 values were converted to 1 cm path length O.D. 600 using a standard curve.

### Bioinformatics analysis

BLAST searches of the genome sequence of *B. cenocepacia *strain J2315 were performed with the *B. cenocepacia *Blast Server at Sanger Institute http://www.sanger.ac.uk/cgi-bin/blast/submitblast/b_cenocepacia. J2315 belongs to the same clonal lineage as strain K56-2 [32]. Gene clusters were visualized with Artemis software [33] and VectorNTI software (Invitrogen). PWM scores were calculated manually [25] (Additional file 2) as described by Hertz and Stormo [34] and Schnieder and Stephens [35]. Identification of binding sites using this PWM was achieved using the Target Explorer [36]. For TCOFFEE analysis [37] the default substitution matrix was used, with a gap opening penalty of -10 and a gap extension penalty of -1.

### Molecular Biology techniques

Recombinant DNA techniques were carried out as previously described [38]. DNA ligase (New England Biolabs) was used as recommended by the manufacturers. *E. coli *DH5α cells were transformed using the calcium chloride protocol [39] and electroporation was used for transformation of *E. coli *SY327 cells [40]. Reporter plasmids were constructed in *E. coli *and conjugation into *B. cenocepacia *K56-2 was accomplished by triparental mating [41] with *E. coli *DH5α carrying the helper plasmid pRK2013 [42]. DNA was amplified using a PTC-221 DNA engine (MJ Research) or an Eppendorf Mastercycler ep gradient S thermal cycler with Taq DNA polymerase, Phusion High-Fidelity PCR Kit or Proofstart DNA polymerase (Qiagen) (New England Biolabs). Amplification conditions were optimized for each primer pair and are available upon request. PCR products and plasmids were purified with QIAquick purification kit (Qiagen) and QIAprep Miniprep kit (Qiagen), respectively.

### RNA isolation methods and RT-PCR analysis

For RNA isolation, bacteria were grown in LB supplemented with 1 mM PA. Cells were harvested during early log phase (O.D. 600 = 0.3) and lysed in TE buffer pH 8.0 containing 400 μl/ml lysozyme for 5 minutes at room temperature. RNA was recovered with the RNeasy Mini kit (Qiagen), and samples eluted into (Diethyl Pyrocarbonate) DEPC treated water. Total RNA was visualized in a 1% agarose gel in TAE buffer. Residual DNA was removed by on column treatment with DNase I (15 min, room temperature), in DNase buffer (Qiagen). The RNA was then used as a template in reverse transcription (RT) or stored at -20°C until use. Reverse transcription was performed by SuperScript RT First-Strand synthesis using relevant gene specific primers (Additional file 1). The resultant cDNA was PCR amplified using gene specific primers (Additional file 1), and the conditions optimized for each reaction. For every PCR, the appropriate controls with water and RNA in the absence of RT were included to ensure that the amplicons obtained were a result of cDNA and not of contaminating genomic DNA.

### Construction of insertional mutant *BCAL0210 *of *B. cenocepacia *K56-2

*BCAL0210 *was disrupted using single crossover mutagenesis with plasmid pGPÙTp, a derivative of pGP704 that carries the *dhfr *gene flanked by terminator sequences [27]. Briefly, an internal 300-bp fragment of *BCAL0210 *was PCR amplified using appropriate primers (Additional file 1). The PCR-amplified was digested with *Xba*I and *EcoR*I respectively, cloned into the *Xba*I and *EcoR*I digested vector and maintained in *E. coli *SY327. The resulting plasmids (Table 1) were conjugated into *B. cenocepacia *strain K56-2 by triparental mating. Conjugants that had the plasmid integrated into the K56-2 genome were selected on LB agar plates supplemented with Tp 100 μg/ml and Gm 50 μg/ml. Integration of the suicide plasmids was confirmed by colony PCR, using primer SC025 that anneals to the R6K origin of replication of pGPÙTp, and primers upstream of the expected site of insertion (Additional file 1). All mutant strains were confirmed by sequencing PCR-amplified DNA fragments containing the insertion site.

### Construction of *eGFP *translational fusion plasmids

To create pJH1, digestion with *Xba*I/*Nde*I of pSCrhaB4 resulted in a 784 bp fragment containing *eGFP*, which was cloned into the same sites in pAP20 [9] such that *eGFP *is under control of the constitutive *dhfr *promoter. *E. coli *transformants were selected with 20 μg/ml chloramphenicol. The plasmid was conjugated into *B. cenocepacia *K56-2 by tri-parental mating with *E. coli *helper strain containing plasmid pRK2013. As *B. cenocepacia *is intrinsically resistant to Gm, in all conjugations Gm was added to the final transfer to eliminate donor *E. coli*. To create pJH2, pJH1 was then PCR amplified using divergently oriented primers (Additional file 1) containing multiple restriction sites on the 5' ends such that the self-ligated product of the reaction has a multiple cloning site in place of the original promoter. Growth rates for *B. cenocepacia *K56-2 with or without pJH2 were similar (data not shown). DNA fragments corresponding to *paaZ *from -420 to +90 (510 bp), *paaA *from -396 to +84 (480 bp), and *paaH *from -327 to +72 (399 bp) of *B. cenocepacia *K56-2 chromosomal DNA were amplified and cloned into pJH2 to create pJH6, pJH7, and pJH8 respectively.

### Construction of site directed plasmid mutants

The plasmids pJH10, pJH11 and pJH12 were constructed by plasmid PCR mutagenesis to contain mutations in the entire, left or right region of the conserved IR in the *paaA *core promoter. Appropriate phosphorylated primers (Additional file 1) were used to divergently amplify template pJH7 (containing the *paaA *promoter), and each contained mismatch mutations on their 5' ends. Plasmids were self-ligated, transformed into *E. coli *DH5α and then conjugated into *B. cenocepacia *wild type. Mutations were verified by sequence analysis (The Centre for Applied Genomics, Toronto).

### Nucleotide accession number

The nucleotide sequence of translational fusion vector pJH2 is deposited in GenBank under accession no. FJ607244.

## Authors' contributions

JNRH participated in the design of the study, carried out the majority of experiments and wrote the manuscript. RAMB was involved in plasmid construction, carried out some reporter analysis and critically read the manuscript. STC participated in the design and coordination of the study and final edition of the manuscript. All authors read and approved the final manuscript.

## Supplementary Material

Additional file 1 Primers used in this study. Click here for file

Additional file 2 **Position Weight Matrix Calculations**. A) The sequences used to generate the matrix of the conserved inverted repeat from the *paaA, paaH, paaZ, paaF *and *BCAL0211 *genes. B) The sum the occurrence of nucleotides at each position. C) The formulas used to generate the PWM, modified from [25] p(*b, i*) = corrected probability of base b in position i; f(*b, i*) = counts of base b in position i; N = number of sites; p(*b*) = background probability of base *b *in *B. cenocepacia *J2315 genome as follows: p(A) or p(T) = 0.1665; p(C) or p(G) = 0.335; W(*b, i*) = PWM value of base *b *in position *i*. D) Resulting position weight matrix. Click here for file

Additional file 3 **Position Weight Matrix scores in a genomic scan of *B. cenocepacia***. The position weight matrix calculated in Additional file 2 was used to scan the genome of *Burkholderia cenocepacia *K56-2. Genome co-ordinate is from the annotated sequence [4]. Click here for file
