# Supplementary material for: Regulation of phenylacetic acid degradation genes of Burkholderia cenocepacia K56-2
Source: BMC Microbiol. 2009 Oct 18;9:222. doi: 10.1186/1471-2180-9-222 (PMC2770484; doi:10.1186/1471-2180-9-222)
Supplement: Additional file 1 — Primers used in this study. [file 1471-2180-9-222-S1.PDF]

Additional file 1. Primers used in this study

| Name | Sequence | Purpose |
|---|---|---|
| SC1 | AACACCCGGGATGGTGAGCAAGGGCGAG | forward primer for amplifying *eGFP* |
| SC 2 | CCGCTCTAGATTACTTGTACAGCTCGTCCA | reverse primer for amplifying *eGFP* |
| SC 3 | GGCGTAGAGGATCTGCTCATGTTTG | forward external primer to check insertion in pAP20 / pJH2 |
| SC 4 | GCTACTGCCGCCAGGCAAATTCTGT | reverse external primer to check insertion in pAP20 / pJH2 |
| SC 11 | GCGCAGATCTCCCGGGCGTACTCATATGCCATGGCTCG AGTAAGCTGTCAAACA | pJH2 forward |
| SC 12 | GCGCAGATCTGGTACCCGTACTGGATCCGAATTCGTG AGCAAGGGCGAGGAGCT | pJH2 reverse |
| SC 23 | CCGTAGGTGGCATCGCCCTC | external promoter sequencing primer |
| SC 30 | GCGTCAGATCTTCGAGATCGTGCTCGACGGCAAG | $P_{BCAL0211}$ forward |
| SC 31 | GCGGTGAATTCGACGACGCGCTCGTCGCGTC | $P_{BCAL0211}$ reverse |
| SC 38 | AGTTAGAATTCGGCGAACGGGCTCCAGTACCCG | $P_{paaZ}$ forward |
| SC 39 | AGTCTGAAGATCTCGGCTTGCGGATCTCGCGGAC | $P_{paaZ}$ reverse |
| SC 43 | AGTCTGAAGATCTAGACGATCTGACGCACGGTTCG | $P_{paaA}$ forward |
| SC 44 | AGTTAGAATTCATCGAACCGCGTCTGCTCGGG | $P_{paaA}$ reverse |
| SC 45 | AGTCTGAAGATCTAGCGCACGTGACGACGCGTG | $P_{paaH}$ forward |
| SC 46 | AGTTAGAATTCGCCGATCACGCCGACGACGG | $P_{paaH}$ reverse |
| SC 51 | ATTATTCTAGACGAGCACGTCGATGTCCGATCTC | BCAL0210 internal fragment forward |
| SC 52 | ATTATGAATTCCGAGCACGATTTCACGCTGCGC | BCAL0210 internal fragment reverse |
| SC 53 | TAACGGTTGTGGACAACAAGCCAGGG | Colony screening vector primer pGPomegaTP |
| SC 66 | ACATCTATGGTTTATACTTCGCACACACTCAACCG | Forward divergent mutagenic primer (HPLC pure 5' P) |
| SC 67 | ATCTCAGTAATAAATTTGTTGTGGATTTGCGGCGGG | Reverse divergent mutagenic primer (HPLC pure 5' P) |
| SC 68 | CGGCTGAAGCACTGCACGCC | sequencing primer for pJH series plasmids |
| SC 72 | GGTCGGTTGGTTTATACTTCGCACAC | Forward divergent NON mutagenic primer match with p67 |
| SC 73 | GGTCGGTTAATAAATTTGTTGTGG | Reverse divergent NON mutagenic primer match with p66 |
| SC 108 | ACCCCGACCTGAAATGGAACGAAG | RT PCR *paaA -> paaB* |
| SC 109 | GTCGGATGACGGTAGATCTTGTCG | RT PCR *paaB -> paaA* |
| SC 110 | CCTCGACCACAAGCATTGCGGC | RT PCR *paaB -> paaC* |
| SC 111 | GTGCGGCAGTTCGGCGAGCG | RT PCR *paaC -> paaB* |
| SC 112 | GACTACCTGATGCCGTACACACG | RT PCR *paaC -> paaD* |
| SC 113 | TCTCGATCCGGTGCGGCGGC | RT PCR *paaD -> paaC* |
| SC 114 | GGACGACCGACTGGATTACGCAG | RT PCR *paaD -> paaE* |
| SC 115 | ACGCGAAGTTCGAGAAGCGGCC | RT PCR *paaE -> paaD* |
| SC 123 | CGTGCAAGGGCGGCGTGTGC | RT PCR *paaE -> BCAL0211* |
| SC 124 | GCGTTCCAGTCGGGAATCTGGTC | RT PCR *BCAL0211 -> paaE* |
| SC 125 | GGGTCAGCAACACGATCAAGAGC | RT PCR *BCAL0211-> BCAL0210* |
| SC 126 | CACCCTCGACCTCGGCGATG | RT PCR *BCAL0210-> BCAL0211* |
