# Supplementary material for: Regulation of phenylacetic acid degradation genes of Burkholderia cenocepacia K56-2
Source: BMC Microbiol. 2009 Oct 18;9:222. doi: 10.1186/1471-2180-9-222 (PMC2770484; doi:10.1186/1471-2180-9-222)
Supplement: Additional file 2 — Position Weight Matrix Calculations. A) The sequences used to generate the matrix of the conserved inverted repeat from the paaA, paaH, paaZ, paaF and BCAL0211 genes. B) The sum the occurrence of nucleotides at each position. C) The formulas used to generate the PWM, modified from [25] p(b, i) = corrected probability of base b in position i; f(b, i) = counts of base b in position i; N = number of sites; p(b) = background probability of base b in B. cenocepacia J2315 genome as follows: p(A) or p(T) = 0.1665; p(C) or p(G) = 0.335; W(b, i) = PWM value of base b in position i. D) Resulting position weight matrix. [file 1471-2180-9-222-S2.PDF]

Additional File 2

A

	Position														
**Sequence**	**1**	**2**	**3**	**4**	**5**	**6**	**7**	**8**	**9**	**10**	**11**	**12**	**13**	**14**	**15**
paaA	A	C	C	G	A	C	C	G	G	T	C	G	G	T	T
paaH	A	C	C	G	A	C	C	G	G	T	C	G	G	T	T
paaZ	A	C	C	A	A	C	C	G	G	T	C	G	G	T	T
paaF	A	C	C	A	A	C	C	G	G	T	C	G	G	T	T
BCAL0211	T	C	C	A	G	C	C	G	G	C	C	G	G	C	G

B

	Position														
**Nucleotide**	**1**	**2**	**3**	**4**	**5**	**6**	**7**	**8**	**9**	**10**	**11**	**12**	**13**	**14**	**15**
**A**	4	0	0	3	4	0	0	0	0	0	0	0	0	0	0
**G**	0	0	0	2	1	0	0	5	5	0	0	5	5	0	1
**C**	0	5	5	0	0	5	5	0	0	1	5	0	0	1	0
**T**	1	0	0	0	0	0	0	0	0	4	0	0	0	4	4

C

corrected probability calculation

$$p(b,i) = \frac{f(b,i) + \sqrt{N} \cdot p(b)}{\sqrt{N} + N}$$

PWM conversion

$$W(b,i) = \log_2\left(\frac{p(b,i)}{p(b)}\right)$$

D

	Position														
**Nucleotide**	**1**	**2**	**3**	**4**	**5**	**6**	**7**	**8**	**9**	**10**	**11**	**12**	**13**	**14**	**15**
**A**	1.86	-1.69	-1.69	1.48	1.86	-1.69	-1.69	-1.69	-1.69	-1.69	-1.69	-1.69	-1.69	-1.69	-1.69
**G**	-1.69	-1.69	-1.69	0.19	-0.47	-1.69	-1.69	1.25	1.25	-1.69	-1.69	1.25	1.25	-1.69	-0.47
**C**	-1.69	1.25	1.25	-1.69	-1.69	1.25	1.25	-1.69	-1.69	-0.47	1.25	-1.69	-1.67	-0.47	-1.69
**T**	0.19	-1.69	-1.69	-1.69	-1.69	-1.69	-1.69	-1.69	-1.69	1.86	-1.69	-1.69	-1.69	1.86	1.86
