# Supplementary material for: Regulation of phenylacetic acid degradation genes of Burkholderia cenocepacia K56-2
Source: BMC Microbiol. 2009 Oct 18;9:222. doi: 10.1186/1471-2180-9-222 (PMC2770484; doi:10.1186/1471-2180-9-222)
Supplement: Additional file 3 — Position Weight Matrix scores in a genomic scan of B. cenocepacia. The position weight matrix calculated in Additional file 2 was used to scan the genome of Burkholderia cenocepacia K56-2. Genome co-ordinate is from the annotated sequence [4]. [file 1471-2180-9-222-S3.PDF]

Additional File 3. Scores from a scan of *B. cenocepacia* J2315 genomic sequence using a Position Weight Matrix.

	Genome coordinate	Score	Downstream CDS	Name / Description	Location from predicted start
Chromosome 1	443094	22.03	PaaZ, PaaF	*paaZ, paaF*	-45, -70
	244306	20.74	PaaA	*paaA*	-44
	25578	17.37	BCAL0023	putative branched-chain amino acid ABC transporter ATP-binding protein	-235
	939333	17.37	BCAL0867	*yjgF*	-734
Chromosome 2	1910367	20.74	PaaH	*paaH*	-42
	781968	18.41	BCAM0706	Ton B dependant receptor (pseudogene) Putative nicotinamide mononucleotide transporter (next in operon)	-18
	2306124	18.41	BCAM2071	Conserved hypothetical protein	-189
Chromosome 3	None				
Plasmid	None				
